# The relationship between mental fatigue, depression, and cognition in Graves’ disease

**DOI:** 10.1530/ETJ-23-0040

**Published:** 2023-07-12

**Authors:** Birgitta Johansson, Mats Holmberg, Simon Skau, Helge Malmgren, Helena Filipsson Nyström

**Affiliations:** 1 Department of Clinical Neuroscience, Sahlgrenska Academy, Institute of Neuroscience and Physiology, University of Gothenburg, Gothenburg, Sweden; 2 Department of Neurology, Sahlgrenska University Hospital, Gothenburg, Sweden; 3 ANOVA, Karolinska University Hospital, Norra Stationsgatan 69, Stockholm, Sweden; 4 Institute of Medicine, Sahlgrenska Academy, University of Gothenburg, Gothenburg, Sweden; 5 Wallenberg’s Centre of Molecular and Translational Medicine, Region Västra Götaland, Sweden; 6 Department of Pedagogical, Curricular and Professional Studies, Faculty of Education, University of Gothenburg, Gothenburg, Sweden; 7 Department of Endocrinology, Sahlgrenska University Hospital, Gothenburg, Sweden

**Keywords:** Graves’, mental fatigue, depression, cognitive function

## Abstract

**Objective:**

Mental fatigue, depression, anxiety, and cognitive complaints are common in Graves’ disease (GD). Our aims were to assess the relationship between these variables in patients with GD during both hyperthyroidism and a long stable euthyroidism.

**Methods:**

A prospective longitudinal case-control study where 65 premenopausal women diagnosed with GD and 65 matched controls were assessed twice with 15 months in between. The first visit for patients was in overt hyperthyroidism and the second after treatment.

**Results:**

During the hyperthyroid phase, mental fatigue, depression, and anxiety were significantly increased for GD patients compared to controls (all *P* < 0.001). Among GD patients, 89% reported mental fatigue and among controls 14%. No difference in cognitive tests was found. After 15 months, significant improvements for GD patients after treatment were found for the items of mental fatigue, depression, and anxiety (all *P* < 0.001), but these were unchanged in controls. GD patients reported residual mental fatigue (38%), 23% without depression, and 15% mental fatigue combined with depression. Self-reported cognitive complaints were pronounced while cognitive tests did not reveal any deficiencies.

**Conclusion:**

Mental fatigue and emotional distress are common in the hyperthyroid phase. These improve with treatment but are still more common in GD patients after 15 months of therapy than in controls. The residual mental fatigue is shown to be a phenomenon distinct from depression in this study. This indicates the importance of assessing mental fatigue in GD patients and underlines the need for rehabilitation and healthcare support as fatigue will have consequences for work ability.

## Introduction

Untreated hyperthyroid patients with Graves’ disease (GD, autoimmune hyperthyroidism) often report fatigue, cognitive problems, and emotional distress. Once the patients are regarded as clinically recovered and euthyroid, the majority will report no residual problems. However, some will still struggle with a reduced quality of life (QoL), fatigue, depression, anxiety, and cognitive complaints and will experience problems with returning to work (1, 2, 3, 4, 5, 6, 7). In GD patients with a previous ability to manage a full-time job, one-third were unable to resume the same work ability after treatment (6, 7, 8, 9, 10, 11). The cognitive problems reported, primarily memory and concentration deficits, are seldom captured with standard neuropsychological tests (10, 12), while more demanding tests have shown altered decision-making and attention when suffering from hyperthyroidism compared to controls (13, 14). There are contradictory results regarding cognitive tests at follow-up (1, 14). In comparison, it has also been difficult to detect cognitive deficits after mild traumatic brain injuries using standard neuropsychological tests, with the result that cognitive difficulties can go undetected, even if the patient has reported fatigue and problems with executive function (15, 16, 17).

The origin of acute and residual symptoms and the relationship with thyroid dysfunction remain unknown (9), along with the way in which, from a clinical perspective, these symptoms interfere with everyday life. Mental fatigue is known to reduce work ability in other clinical contexts (18), and it is, therefore, necessary to screen for it routinely. Mental fatigue and depression should be considered separately as the treatment options differ depending on the condition.

The aim of this study was to assess mental fatigue, depression, anxiety, and cognitive function in GD patients in the hyperthyroid phase and after 15 months of treatment, with the intention to elucidate factors that are not primary targets in the treatment of GD and which need to be addressed during rehabilitation. The following three hypotheses have received support: (i) mental fatigue, depression, anxiety, and cognitive disturbances are pronounced in GD patients in the acute phase; (ii) residual mental fatigue after 15 months of treatment is still a common symptom; and (iii) standard cognitive tests do not capture cognitive complaints experienced subjectively.

## Methods

### Study subjects and study design

The CogThy study, previously described in detail by Holmberg *et al.* (19) was a prospective, longitudinal case–control study that included 65 premenopausal women diagnosed with GD for the first time (in total, 116 were invited to participate in the study). They were recruited from the Thyroid Units at Sahlgrenska University Hospital, Gothenburg (*n* = 64), and Kungälv’s Hospital, Kungälv (*n* = 1) in Sweden. The median duration of symptoms from debut to diagnosis was 4.0 months. Inclusion criteria were as follows: (i) premenopausal, (ii) free thyroxine ≥ 50 pmol/L (reference range 12–22) and/or total triiodothyronine ≥ 6.0 pmol/L (reference range 1.3–3.1), and (iii) positive thyroid-stimulating hormone receptor antibodies or a diffuse uptake on technetium scintigraphy. Exclusion criteria were as follows: (i) pregnancy, (ii) other serious diseases (endocrine disease, heart failure, respiratory failure, malignancy, psychosis, active thyroid-associated ophthalmopathy), (iii) inability to follow the study protocol, (iv) past, present, or expected use of steroids within the next 15 months, and (v) patients with contraindications for magnetic resonance imaging or with amiodarone-induced GD. One patient was later excluded because of the onset of menopause, leaving 64 for the analyses. Patients that were likely to need steroids for ophthalmopathy (thyroid-associated ophthalmopathy; TAO) in the next 15 months were excluded. Clinical activity score (CAS) of 61 participants were at baseline; 2 (3%) CAS 3, 12 (20%) CAS 2, 15 (25%) CAS 1, and the rest CAS 0. At 15 months, 5 patients had worsened CAS, 5 had unchanged CAS, and 19 had improved CAS compared to baseline. None of the patients with worsened CAS exceeded CAS 3 at 15 months.

Sixty-five matched controls were identified from the Swedish Tax Registry. They were excluded if they had previous or ongoing thyroid disease or thyroid hormone levels outside the normal reference range. The GD and control groups were matched regarding sex, age, smoking status, and education. Mean age did not differ significantly between the groups and, for the GD patients, the mean age was 30.5 years (range 24.5–38.0) and, for the controls, the mean age was 31.5 years (26.5–39.0).

Patients as well as controls underwent an assessment at inclusion and after a mean of 16.4 ± 4.2 (s.d.) months, including questionnaires to evaluate mental fatigue, anxiety, and depression and a neuropsychological assessment. The participants were included from September 2011 to October 2019 at the Department of Endocrine Research, Sahlgrenska University Hospital, Gothenburg, Sweden. Patients were included within 2 weeks after starting treatment with antithyroid drugs (ATD, thiamazole). All patients were initially treated with ATD and when necessary with surgery. At 15 months, 36% had undergone thyroidectomy, one patient was treated with radioactive iodine, 47% were receiving ATD, and 17% had no treatment and were in remission. Due to the high thyroid hormone levels required for inclusion and the high risk for recurrent disease, a larger proportion than reported from other studies was thyroidectomised. Also, surgery is the recommended treatment of choice in Sweden in women < 35 years if an ablative treatment is chosen. Radioactive iodine treatment has also been connected to reduced QoL. For these reasons, radioactive iodine treatment frequency was low (20).

### Ethics

Ethical approval was granted by the Regional Ethical Review Board in Gothenburg, Sweden (Dnr 190-10). The study was conducted in accordance with the Declaration of Helsinki and was registered in the public project database for research and development in Västra Götaland Region, Sweden (https://www.researchweb.org/is/vgr/project/44321). Written consent has been obtained from each patient or subject after full explanation of the purpose and nature of all procedures used.

### Measures

The Mental Fatigue Scale (MFS) is based on extensive clinical research into diseases that affect the brain (20) and has been evaluated for people with acquired brain injury (21). A value above 10 indicates a significant problem with mental fatigue. The higher the value, the greater the problems. The items in the MFS questionnaire include the following: generalized fatigue, fatigue related to mental activities, mental recovery time, concentration and memory problems, slowness of thinking, stress sensitivity, emotional sensitivity and irritability, reduced initiative, light and sound sensitivity, and sleep problems. The questions have a high internal consistency (Cronbach’s alpha of 0.944) (21, 22). For self-assessment of depression and anxiety, the Comprehensive Psychopathological Rating Scale (CPRS) was used (23). Mild depression has been associated with a rating between 6.6 and 9.5, moderate between 10 and 17, and severe ≥ 17.5 (24). The CPRS depression scale is identical to the Montgomery Åsberg Depression Rating Scale (MADRS) except that the rating is doubled in the MADRS (25).

### Neuropsychological tests

The cognitive tests from WAIS-III (26) were digit symbol-coding measuring information processing speed and digit span measuring auditory attention and working memory. Digit span includes repetition of a forward series of random numbers as well as repetition of the numbers in reverse (26). The verbal fluency test measures the ability to generate as many words as possible beginning with a specific letter within 1 min (27). The Trail Making Test A and B measure visual scanning, divided attention, and motor speed (28). The test consists of a series of connect-the-circle tasks, part A with a numerical order of 1 to 25 and part B comprising letters and digits in alternating numerical and alphabetical order, both parts to be completed as quickly as possible. In order to evaluate higher demands such as dual tasks, a series of new tests was constructed with three and four factors, respectively (29). The same number of circles (25) was used in all parts. The alternation between factors was similar to part B except for the fact that months were added in part C and both months and days of the week were added in chronological order in part D. In the latter, the order of letters and digits was changed (A: 1 to 25, B: 1-A-2-B-3-C, C: 1-A-January-2-B-February-3-C-March, D: A-1-January-Monday-B-2-February-Tuesday). Reading speed was measured using a dyslexia reading speed test (30). Participants were instructed to read the text to themselves and to mark the correct word that corresponded to the meaning of the sentence selected from evenly distributed brackets containing three words; this test serves as an evaluation of reading comprehension. The whole text contained 650 words.

### Statistics

For the main analysis, a general linear model with time as a repeated factor and group as a between factor was used. Participants with missing data were not included in the pre-post analysis. Bonferroni–-Holm correction was used for multiple comparison and *t*-test for group comparison. For comparison between subjective data (questionnaires) and objective data (cognitive tests), Z-values were calculated (Z = (mean GD − mean controls)/s.d. controls). SPSS 28.0 (IBM) was used for statistical calculations. Statistical significance was set to 0.05.

## Results

Hyperthyroid baseline data included 64 patients and 64 controls. At 15 months, 53 patients (83%) and 38 controls (59%) were followed up. The reasons for dropout among GD patients and controls were lack of time, not wishing to continue, or migration out of the area. Fewer controls were followed up, as the primary analysis in the CogThy study with magnetic resonance data did not show any longitudinal change in a subset of controls (4).

**Figure 1:**
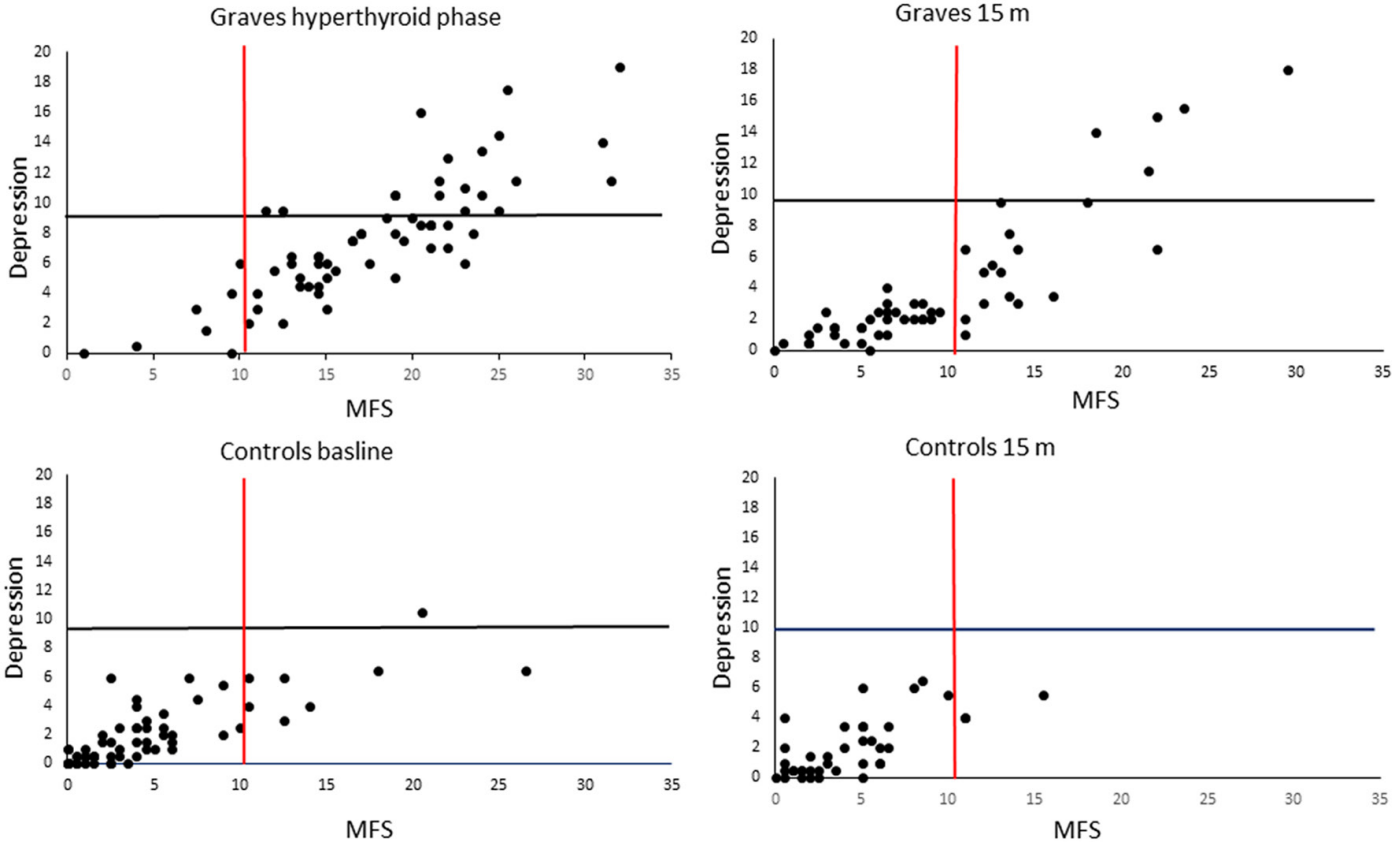
Relationship between mental fatigue and depression in GD patients and controls, in the hyperthyroid phase (64 GD/64 controls) and after 15 months (53 GD/38 controls). The lines indicate cut-off scores for mental fatigue (MFS), indicating fatigue on the right side of the red vertical line and probable depression (CPRS) above the horizontal black line.

In hyperthyroidism, mental fatigue (MFS), depression, and anxiety scores (CPRS) were all significantly higher in GD patients compared to controls (all *P* < 0.001). Among GD patients in the hyperthyroid phase, 34.5% reported mental fatigue with no depression and 54.5% mental fatigue with mild to moderate/severe depression according to cut-off scores. Most of the controls reported no fatigue and no depression (Fig. 1). No significant difference was detected between GD patients and controls for any of the cognitive tests in the hyperthyroid phase (data not shown, except mean values, in Table 1).

**Table 1:** Results from the general linear model with repetition of assessment (time) and comparison between groups as well as interaction effect for time and group. Data are presented as mean (s.d.), and *P*-values.

	Number of*, n*		GD, mean (s.d.)		Controls, mean (s.d.)		*P*-values		
	GD	CON	HP	15 months	0 month	15 months	Time	Interaction	Group
MFS	53	39	17.5 (6.3)	9.5 (6.4)	5.6 (5.5)	4.3 (3.5)	<0.001	<0.001^a^	<0.001
Depression	53	38	7.5 (4.1)	3.9 (4.2)	1.9 (1.9)	2.0 (2.0)	<0.001	<0.001^a^	<0.001
Anxiety	53	38	8.3 (4.2)	5.0 (3.9)	3.0 (2.5)	3.0 (2.4)	<0.001	<0.001^a^	<0.001
TMT A	50	38	28.3 (12.0)	23.8 (8.4)	27.6 (8.1)	25.3 (6.8)	<0.001	0.218	0.839
TMT B	50	38	67.8 (25.1)	62.0 (27.4)	63.6 (24.0)	58.9 (21.0)	0.014	0.778	0.457
TMT C	49	38	63.0 (22.0)	58.7 (24.3)	58.2 (21.5)	58.1 (15.8)	0.194	0.215	0.537
TMT D	49	38	110.7 (36.7)	100.8 (41.1)	111.5 (37.7)	106.5 (42.5)	0.041	0.504	0.676
DSC	50	38	78.4 (13.4)	81.5 (16.1)	80.9 (12.9)	84.1 (11.6)	<0.001	0.672	0.326
Digit span	50	38	15.6 (3.9)	15.0 (3.5)	14.9 (3.6)	16.0 (3.5)	0.340	0.005^a^	0.770
Digit span forward	50	38	9.2 (2.0)	8.9 (1.8)	8.6 (2.3)	9.5 (1.9)	0.125	0.008	0.957
Digit span backward	50	38	6.3 (2.0)	6.3 (1.7)	6.6 (3.2)	6.7 (2.2)	0.954	0.954	0.420
FAS	50	38	42.6 (11.4)	45.5 (11.2)	42.1 (11.1)	45.2 (11.5)	0.954	0.954	0.420
RS Words/s	49	38	3.11 (0.87)	3.08 (0.75)	3.10 (0.82)	3.43 (0.76)	0.007	<0.001^a^	0.355

^a^Indicates that significance remains after the Bonferroni–Holm correction.

CON, controls; DSC, digit symbol coding; FAS, word fluency test; GD, Graves’ disease; HP, hyperthyroid phase; m, months; MFS, Mental Fatigue Scale; RS, reading speed; TMT, trail making test.

The general linear model including between- (GD patients and controls) and within-subject factors (time; hypothyroid phase and 15 months after therapy) revealed significant post-therapy improvements in mental fatigue, depression, anxiety, digit span total and forward, and reading speed (Table 1). The patients reduced their rating on mental fatigue, depression, and anxiety, while for the controls, these were unchanged.

**Figure 2:**
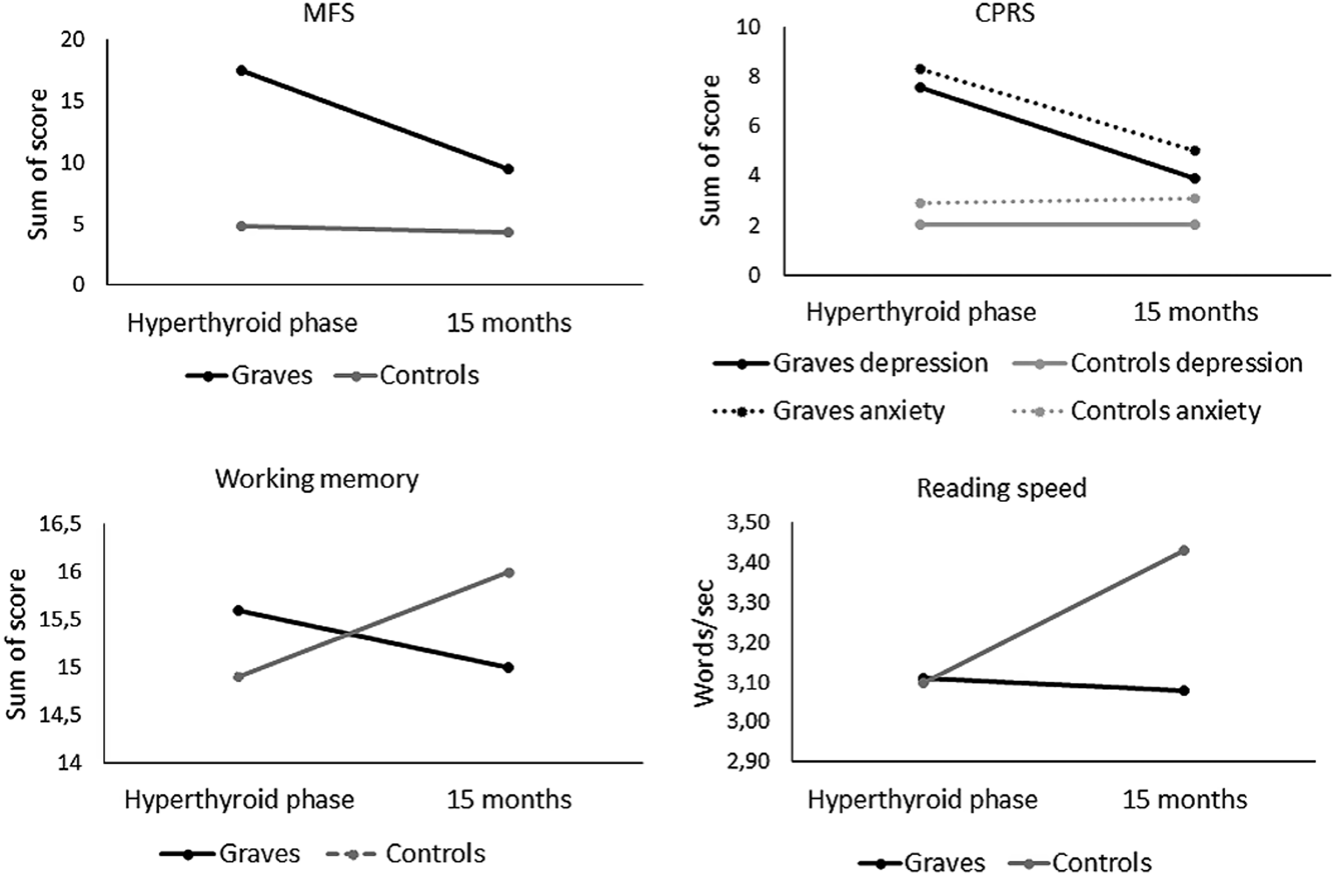
Interaction effects between time (hyperthyroid phase – 15 months) and groups (Graves’ disease and controls). Significant interactions were found for mental fatigue (MFS), depression and anxiety (CPRS) and the cognitive tests, working memory (digit span), and reading speed.

After correction for multiple comparison (Bonferroni–Holm correction), significant post-therapy improvements remained for mental fatigue, depression, anxiety, reading speed, and digit span (Fig. 2 shows the interaction effect). None of the other cognitive tests differed significantly between the GD group and controls.

After 15 months of therapy, 23% of the GD patients reported mental fatigue without depression, 15% reported mental fatigue with mild-to-severe depression, and 62% reported neither fatigue nor depression (Fig. 1).

**Figure 3:**
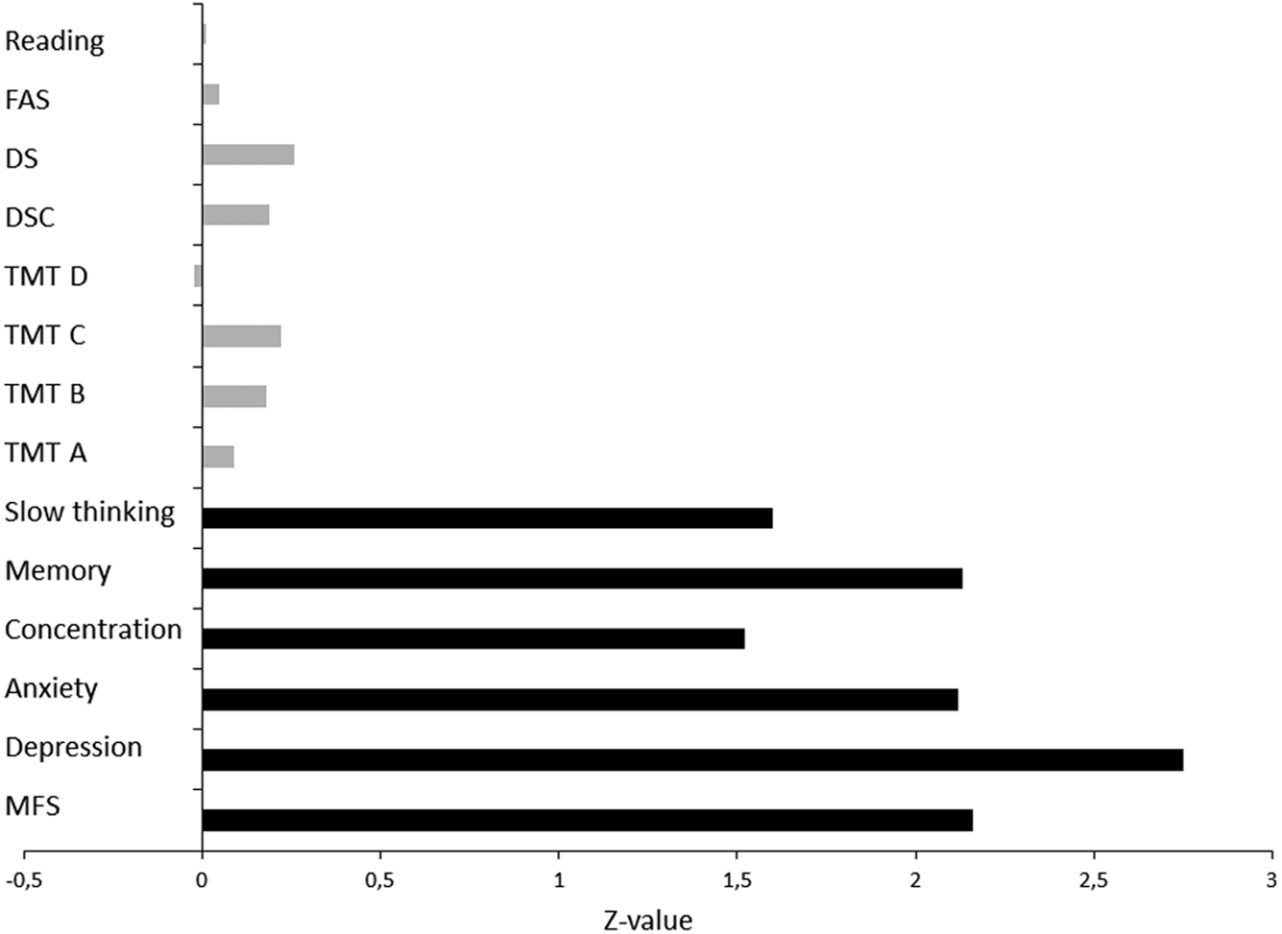
Z-values showing the discrepancy (difference) in the hyperthyroid phase between the GD treatment group and the control group in subjective cognitive ratings (black, items from MFS, and also total sum of scores on the MFS and CPRS, depression and anxiety) and cognitive tests (grey). Cognitive tests; reading speed, word fluency (FAS), working memory (DS), processing speed (DSC), and divided attention (TMT).

Subjective experience of cognitive symptoms reported with items from MFS (concentration, memory, and slowness of thinking) and the results from cognitive tests were compared in the hyperthyroid phase. The Z-values indicate a clear discrepancy between cognitive tests and subjective data (the differences are shown in Fig. 3). Self-reported cognitive complaints were more pronounced compared to results from the cognitive tests performed.

## Discussion

In comparison with the control group, GD patients in the hyperthyroid phase reported a significantly higher rating on mental fatigue and emotional complaints (depression, anxiety), while no difference was detected for any of the cognitive tests. After 15 months of treatment, significant improvements were found for MFS (mental fatigue) and CPRS (depression, anxiety) in the GD patients, while the controls remained on a lower level with no change over time. Only 14% of the controls reported significant mental fatigue at baseline compared to 89% for GD patients. At follow-up, 38% of GD patients still reported residual mental fatigue, 23% mental fatigue only, and 15% mental fatigue and depression; 7% of controls reported mental fatigue slightly above cut-off and no depression. This shows that mental fatigue and depression are separate states. We suggest that mental fatigue needs to be acknowledged and assessed, and also suggest the use of MFS for mental fatigue because it is a promising evaluation option.

There was a clear distinction between experienced self-reported cognitive complaints (memory, concentration, slowness of thinking) and cognitive tests, with only subtle significant changes over time, for working memory and reading speed in comparison with the controls. Our study is in agreement with other studies reporting emotional complaints and fatigue for those suffering from GD in the hyperthyroid phase (10, 11). Our study also conforms with other studies reporting that a subgroup of patients suffers residual subjective cognitive problems (5, 6, 7, 12), with no or subtle cognitive impairments on neuropsychological tests (10, 12, 13, 14). A similar discrepancy between cognitive tests and subjective cognitive function has previously been shown for burn-out patients in comparison to controls (31). It could be argued that neuropsychological tests for cognitive function should be the golden standard. However, these tests are developed to identify focal brain damage (32) and are not intended to predict function in everyday life (33). The cognitive deficits reported in this study were not detected with traditional neuropsychological tests. Mental fatigue with co-occurring cognitive complaints is common in many acquired brain disorders (34) and there are currently no neuropsychological methods for assessing this. MFS (35) is based on concrete questions that together cover mental fatigue syndrome. No comparable method is available. For those suffering from mental fatigue, there is a discrepancy between function as measured with cognitive tests and the ability to maintain the continuous performance of tasks and work throughout the day without total mental exhaustion (36). There is a reduction in endurance in terms of mental activity, and it has been suggested that brain function is less efficient in the case of mental fatigue (37, 38, 39, 40, 40). The perceived lack of energy is suggested to be a pathological brain state (41), and the evaluation of mental fatigue using the MFS assessment scale is considered relevant for appraising work ability (18, 42). Therefore, it is important to evaluate mental fatigue as a separate issue.

There is an increasing awareness of fatigue in GD (7, 10), which we have also reported here, with 89% suffering from mental fatigue during the hyperthyroid phase and a clear discrepancy of 14% between this group and the population-based control. After 15 months of therapy, 38% still suffered from mental fatigue. Some of the participants in the GD group also suffered from depression. It could be difficult to differentiate between mental fatigue and depression as symptom overlaps exist in the questionnaires (in this study, the items, concentration problems, initiative, and decreased sleep are the same items and are rated in the same way in both MFS and CPRS, depression). This overlap is also referred to and discussed for other disorders (acquired brain injuries), and fatigue and depression are suggested to be treated as separate states (43, 44, 45). We agree with these findings. It is to be noted that the result here is based on assessment scales and cut-off scores and not on clinical assessments. In the clinic, a thorough examination is always essential, and the overlap in questionnaires between mental fatigue and depression must be further delineated. Most clinicians are well aware of the clinical manifestations of depression, and medical and psychological treatment methods are well-established for depression. Less is known about fatigue and there is a limited understanding of mental fatigue in relation to work ability and everyday life. Mental fatigue can therefore remain undetected if not evaluated. Today rehabilitation and treatment options for mental fatigue are scarce. It is possible to ameliorate mental fatigue with stimulating drugs and with behavioural and mindfulness techniques (46, 47, 48, 49, 50, 51). It is also important to help the patient to be aware of residual problems and to initiate them in ways in which to manage their mental fatigue and emotional distress. It is necessary to offer the patient rehabilitation and long-term healthcare support provided by a professional team qualified in several relevant areas.

### Limitations

This study was performed over a long period and not all GD patients wished to participate in the study with only 56 % of the GD accepting, for the following reasons: (i) the inclusion rate was slower than expected, (ii) COVID-19 pandemic, and (iii) some patient dropouts at follow-up. The strengths of the study are as follows: (i) the inclusion criteria, (ii) a well-characterized group, and (iii) a matched control group.

In conclusion, mental fatigue and cognitive, and emotional complaints were common in GD patients in the hyperthyroid phase. These symptoms were detected using questions included in MFS rather than standard neuropsychological tests. Notwithstanding, about one-third of the patients reported mental fatigue primarily and some fatigue together with depressive symptoms after 15 months of treatment.

We suggest that mental fatigue should be included in the assessment for GD patients as it may reduce work ability and QoL. The persistence of long-term symptoms highlights the importance of follow-up offering a rehabilitation option.

## Declaration of interest

The authors declares that they have no relevant or material financial interests that relate to the research described in this paper.

## Funding

The study was financed by grants from the Swedish State under the agreement between the Swedish government and the county councils, the ALF-agreement (ALFGBG-717311, ALFGBG-790271), the Healthcare Board, Region Västra Götaland (Hälso- och sjukvårdsstyrelsen), Sahlgrenska University Hospital research funds, Gothenburg Medical Society, Swedish Medical Society, Swedish Society for Medical Research, Swedish Endocrine Society http://dx.doi.org/10.13039/100005709, The Fredrik and Ingrid Thuring's Foundation, The Iris Grant, The Jeansson's Foundation, The Tore Nilsson's Foundation, The Wilhelm and Martina Lundgren's Foundation, The Pharmacist Hedberg's Foundation, The Anna-Lisa and Bror Björnsson's Foundation, The Adlerbert Research Foundation, and The Åke Wiberg's Foundation. The Region Västra Götaland, Sweden, is acknowledged for generous support.

## Statement of ethics

Ethical approval was granted by the Regional Ethical Review Board in Gothenburg, Sweden (Dnr 190-10). The study was conducted in accordance with the Declaration of Helsinki and was registered in the public project database for research and development in Västra Götaland Region, Sweden (https://www.researchweb.org/is/vgr/project/44321). Written consent has been obtained from each patient or subject after full explanation of the purpose and nature of all procedures used.

## References

[1] Lillevang-Johansen M Petersen I Christensen K Hegedüs L & Heiberg Brix TH. Is previous hyperthyroidism associated with long-term cognitive dysfunction? A twin study. Clinical Endocrinology 2014 80 290–295. (10.1111/cen.12255) 23721651

[2] Ritchie M & Yeap BB. Thyroid hormone: influences on mood and cognition in adults. Maturitas 2015 81 266–275. (10.1016/j.maturitas.2015.03.016) 25896972

[3] Bunevicius R & Prange AJ. Psychiatric manifestations of Graves’ Hyperthyriodism. Theraphy in Practice 2006 20 897–909. (10.2165/00023210-200620110-00003) 17044727

[4] Holmberg M Malmgren H Heckemann RA Johansson B Klasson N Olsson E Skau S Starck G & Filipsson Nyström H. A longitudinal study of medial temporal lobe volumes in Graves' disease. Journal of Clinical Endocrinology and Metabolism 2021 dgab808. (10.1210/clinem/dgab808) PMC8947220 34752624

[5] Abraham-Nordling M Törring O Hamberger B Lundell G Tallstedt L Calissendorff J & Wallin G. Graves´disease: a long-term quality-of-life follow up of patients randomized to treatment with antithyroid drugs, radioiodine, or sugery. Thyroid 2005 15 1279–1286. (10.1089/thy.2005.15.1279) 16356093

[6] Cramon P Winther KH Watt T Bonnema SJ Bue Bjorner JB Ekholm O Groenvold M Hegedüs L Feldt-Rasmussen U & Krogh Rasmussen ÅK. Quality-of-life impairments persist six months after treatment of graves' hyperthyroidism and toxic nodular goiter: a prospective cohort study. Thyroid 2016 26 1010–1018. (10.1089/thy.2016.0044) 27370744

[7] Fahrenfort JJ Wilterdink AML & van der Veen EA. Long-term residual complaints and psychosocial sequelae after remission of hyperthyroidism. Psychoneuroendocrinology 2000 25 201–211. (10.1016/s0306-4530(9900050-5) 10674283

[8] Wallace JE & MacCrimmon DJ Hyperthyroidism: Cognitive and Emotional Factors, pp 323–343: New York, NY, USA: Springer, 1990.

[9] Eslami-Amirabadi M & Ahmad Sajjadi S. The relation between thyroid dysregulation and impaired cognition/behaviour: an integrative review. Journal of Neuroendocrinology 2020 33 e12948. (10.1111/jne.12948) PMC8087167 33655583

[10] Vogel A Elberling TV Hørding M Dock J Rasmussen AK Feldt-Rasmussen U Perrild H & Waldemar G. Affective symptoms and cognitive functions in the acute phase of Graves' thyrotoxicosis. Psychoneuroendocrinology 2007 32 36–43. (10.1016/j.psyneuen.2006.09.012) 17097812

[11] Möller MC Bartfai A Nygren de Boussard C Flöter Rådestad AF & Calissendorff J. High rates of fatigue in newly diagnosed Graves' disease. Fatigue: Biomedicine, Health and Behavior 2014 2 153–162. (10.1080/21641846.2014.935279)

[12] Riguetto CM Neto AM Tambascia MA & Zantut-Wittmann DE. The relationship between quality of life, cognition, and thyroid status in Graves' disease. Endocrine 2019 63 87–93. (10.1007/s12020-018-1733-y) 30173328

[13] Yuan L Tian Y Zhang F Ma H Chen X Dai F & Wang K. Decision-making in patients with hyperthyroidism: a neuropsychological study. PLoS One 2015 10 e0129773. (10.1371/journal.pone.0129773) PMC4474662 26090955

[14] Yuan L Zhang Y Luan D Xu X Yang Q Zhao S & Zhou Z. Reversible affective symptoms and attention executive control network impairment following thyroid function normalization in hyperthyroidism. Neuropsychiatric Disease and Treatment 2019 15 3305–3312. (10.2147/NDT.S227386) 32063707 PMC6884974

[15] Bigler ED. Neuropsychology and clinical neuroscience of persitent postconcussive syndrome. Journal of the International Neuropsychological Society 2008 14 1–22. (10.1017/S135561770808017X) 18078527

[16] Cicerone K Levin H Malec J Stuss D & Whyte J. Cognitive rehabilitation interventions for executive function: moving from bench to bedside in patients with traumatic brain injury. Journal of Cognitive Neuroscience 2006 18 1212–1222. (10.1162/jocn.2006.18.7.1212) 16839293

[17] Chan RCK Shumb D Toulopoulou T & Chend EYH. Assessment of executive functions: review of instruments and identification of critical issues. Archives of Clinical Neuropsychology 2008 23 201–216. (10.1016/j.acn.2007.08.010) 18096360

[18] Johansson B. Screening method for assessment of work ability for patients suffering from mental fatigue. Frontiers in Behavioral Neuroscience 2022 16 869377. (10.3389/fnbeh.2022.869377) PMC9237561 35775012

[19] Holmberg MO Malmgren H Heckemann RA Johansson B Klasson N Olsson E Skau S Starck G & Filipsson Nyström H. Structural brain changes in hyperthyroid Graves’ disease: protocol for an ongoing longitudinal, casecontrolled study in Göteborg, Sweden— the CogThy project. BMJ Open 2019 9 e031168. (10.1136/bmjopen-2019-031168) PMC6858258 31685507

[20] Lindqvist G & Malmgren H. Organic mental disorders as hypothetical pathogenetic processes. Acta Psychiatrica Scandinavica. Supplementum 1993 373(Supplement 373) 5–17. (10.1111/j.1600-0447.1993.tb05611.x) 8372702

[21] Johansson B & Rönnbäck L. Evaluation of the Mental Fatigue Scale and its relation to Cognitive and Emotional Functioning after traumatic Brain Injury or Stroke. International Journal of Physical Medicine and Rehabilitation 2014 2 182.

[22] Johansson IB Starmark A Berglund P Rödholm M & Rönnbäck L. Mental trötthet – subjektivt problem som kan skattas. Lakartidningen 2010 107 2964–2967. (10.4172/2329-9096.1000182) 21213607

[23] Svanborg P & Åsberg M. A new self-rating scale for depression and anxiety states based on the Comprehensive Psychopathological Rating Scale. Acta Psychiatrica Scandinavica 1994 89 21–28. (10.1111/j.1600-0447.1994.tb01480.x) 8140903

[24] Snaith RP Harrop FM Newby DA & Teale C. Grade scores of the Montgomery-Asberg Depression and the Clinical Anxiety Scales. British Journal of Psychiatry 1986 148 599–601. (10.1192/bjp.148.5.599) 3779233

[25] Montgomery SA & Åsberg M. A new depression scale designed to be sensitive to change. British Journal of Psychiatry 1979 134 382–389. (10.1192/bjp.134.4.382) 444788

[26] Wechsler D Wechsler Adult Intelligence Scale, 3rd ed. Stockholm, Sweden: Pearson Assessment, 2004.

[27] Delis DC Kaplan E & Kramer JH. Delis-Kaplan Executive Function System – D-KEFS. San Antonio, TX, USA: The Psychological Corporation, 2001.

[28] Reitan RM & Wolfson D. The Halstead-Reitan neuropsychological Test Battery. Theory and clinical interpretation. Tucson, AZ, USA: Neuropsychology Press, 1985.

[29] Johansson B Berglund P & Rönnbäck L. Mental fatigue and impaired information processing after mild and moderate traumatic brain injury. Brain Injury 2009 23 1027–1040. (10.3109/02699050903421099) 19909051

[30] Läsdiagnos M Läsdiagnos. Lund: Läs och skrivcentrum 2003.

[31] Nelson A Malmberg Gavelin H Boraxbekk C-J Eskilsson T Josefsson M Slunga Järvholm L & Stigsdotter Neely A. Subjective cognitive complaints in patients with stress-related exhaustion disorder: a cross sectional study. BMC Psychology 2021 18 84. (10.1186/s40359-021-00576-9) PMC8132387 34006315

[32] Lezak MD Howieson DB & Loring DW Neuropsychological Assessment4th ed. New York, NY, USA: Oxford University Press, 2004.

[33] Sbordone RJ. Neuropsychological tests are poor at assessing the frontal lobes, executive functions, and neurobehavioral symptoms of traumatically brain-injured patients. Psychological Injury and Law 2010 3 25–35. (10.1007/s12207-010-9068-x)

[34] Johansson B Starmark A Berglund P Rödholm M & Rönnbäck L. A self-assessment questionnaire for mental fatigue and related symptoms after neurological disorders and injuries. Brain Injury 2010 24 2–12. (10.3109/02699050903452961) 20001478

[35] Johansson B. Mental fatigue after mild traumatic brain injury in relation to cognitive tests and brain imaging methods. International Journal of Environmental Research and Public Health 2021 18 5955. (10.3390/ijerph18115955) PMC8199529 34199339

[36] Johansson B & Rönnbäck L. Long-lasting mental fatigue after traumatic brain injury – A major problem most often neglected diagnostic criteria, assessment, relation to emotional and cognitive problems, cellular background, and aspects on treatment. In Traumatic Brain Injury. Ed Sadaka F. Rijeka, Croatia: INTECH, 2014.

[37] Skau S Bunketorp-Käll L Kuhn HG & Johansson B. Mental fatigue and functional near-infrared spectroscopy (fNIRS) – based assessment of cognitive performance after mild traumatic brain injury. Frontiers in Human Neuroscience 2019 13 145. (10.3389/fnhum.2019.00145) 31139065 PMC6527600

[38] Skau S Jonsdottir IH Sjörs Dahlman A Johansson B & Kuhn HG. Exhaustion disorder and altered brain activity in frontal cortex detected with fNIRS. Stress 2021 24 64–75. (10.1080/10253890.2020.1777972) 32510268

[39] Berginström N Nordström P Ekman U Eriksson J Andersson M Nyberg L & Nordström A. Using functional magnetic resonance imaging to detect chronic fatigue in patients with previous traumatic brain injury: changes linked to altered striato-thalamic-cortical functioning. Journal of Head Trauma Rehabilitation 2018 33 266–274. (10.1097/HTR.0000000000000340) 28926483

[40] Wylie GR Dobryakova E DeLuca J Chiaravalloti N Essad K & Genova H. Cognitive fatigue in individuals with traumatic brain injury is associated with caudate activation. Scientific Reports 2017 7 8973. (10.1038/s41598-017-08846-6) 28827779 PMC5567054

[41] Rönnbäck L & Johansson B. Long-lasting pathological mental fatigue after brain injury–A dysfunction in glutamate neurotransmission? Frontiers in Behavioral Neuroscience 2021 15 791984. (10.3389/fnbeh.2021.791984) PMC8841553 35173592

[42] Palm S Rönnbäck L & Johansson B. Long-term mental fatigue after traumatic brain injury and impact on capacity for workemployment status. Journal of Rehabilitation Mededicine 2017 49 228–233. (10.2340/16501977-2190) 28150857

[43] Western E Nordenmark TH Sorteberg W Karic T & Sorteberg A. Fatigue after Aneurysmal subarachnoid hemorrhage: clinical Characteristics and Associated Factors in Patients with good outcome. Frontiers in Behavioral Neuroscience 2021 15 633616. (10.3389/fnbeh.2021.633616) PMC8149596 34054441

[44] Cantor JB Ashman T Gordon W Ginsberg A Engmann C Egan M Spielman L Dijkers M & Flanagan S. Fatigue after traumatic brain injury and its impact on participation and quality of life. Journal of Head Trauma Rehabilitation 2008 23 41–51. (10.1097/01.HTR.0000308720.70288.af) 18219234

[45] Kluger BM Krupp LB & Enoka RM. Fatigue and fatigability in neurologic illnesses. Proposal for a unified taxonomy. Neurology 2013 80 409–416. (10.1212/WNL.0b013e31827f07be) 23339207 PMC3589241

[46] Johansson B Andréll P Rönnbäck L & Mannheimer C. Follow-up after 5.5 years of treatment with methylphenidate for mental fatigue and cognitive function after a mild traumatic brain injury. Brain Injury 2020 34 229–235. (10.1080/02699052.2019.1683898) 31657646

[47] Johansson B Bjuhr H & Rönnbäck L. Mindfulness based stress reduction improves long-term mental fatigue after stroke or traumatic brain injury. Brain Injury 2012 26 1621–1628. (10.3109/02699052.2012.700082) 22794665

[48] Johansson B Wentzel AP Andréll P Mannheimer C & Rönnbäck L. Methylphenidate reduces mental fatigue and improves processing speed in persons suffered a traumatic brain injury. Brain Injury 2015 29 758–765. (10.3109/02699052.2015.1004747) 25794299

[49] Johansson B Carlsson A Carlsson ML Karlsson M Nilsson MKL Nordquist-Brandt E & Rönnbäck L. Placebo-controlled cross-over study of the monoaminergic stabiliser (−)-OSU6162 in mental fatigue following stroke or traumatic brain injury. Acta Neuropsychiatrica 2012 24 266–274. (10.1111/j.1601-5215.2012.00678.x) 25286991

[50] Nguyen S McKay A Wong D Rajaratnam SM Spitz G Williams G Mansfield D & Ponsford JL. Cognitive behavior therapy to treat sleep disturbance and fatigue after traumatic brain injury: a pilot randomized controlled trial. Archives of Physical Medicine and Rehabilitation 2017 98 1508–1517.e2. (10.1016/j.apmr.2017.02.031) 28400181

[51] Grossman P Kappos L Gensicke H D'Souza M Mohr DC Penner IK & Steiner C. MS quality of life, depression, and fatigue improve after mindfulness training: a randomized trial. Neurology 2010 75 1141–1149. (10.1212/WNL.0b013e3181f4d80d) 20876468 PMC3463050

